# Decreased quantitative transport mapping velocity in the middle cerebral artery–supplied temporal lobe in Alzheimer's disease

**DOI:** 10.1002/alz.70540

**Published:** 2025-07-29

**Authors:** Yihao Guo, Tao Liu, Yi Li, Gloria C. Chiang, Weiyuan Huang, Yiying Zhang, Huijuan Chen, Mony J. de Leon, Tracy A. Butler, Yi Wang, Feng Chen, Liangdong Zhou

**Affiliations:** ^1^ Department of Radiology Hainan General Hospital (Hainan Affiliated Hospital of Hainan Medical University) Haikou China; ^2^ Department of Neurology Hainan General Hospital (Hainan Affiliated Hospital of Hainan Medical University) Haikou China; ^3^ Department of Radiology Brain Health Imaging Institute (BHII) Weill Cornell Medicine New York New York USA; ^4^ Department of Radiology Division of Neuroradiology Weill Cornell Medicine New York‐Presbyterian Hospital New York New York USA; ^5^ Department of Radiology MRI Research Institute (MRIRI) Weill Cornell Medicine, United States New York New York USA

**Keywords:** Alzheimer's disease, arterial territories blood supply, cognitive functions, Granger causality analysis, interstitial fluid dynamics, multi‐post labeling delay arterial spin labeling (mPLD‐ASL), neurofluids, perfusion velocity, quantitative transport mapping

## Abstract

**INTRODUCTION:**

Reduced blood perfusion has been observed in patients with Alzheimer's disease (AD), but the patterns of blood perfusion changes during AD progression remain insufficiently explored.

**METHODS:**

Quantitative transport mapping (QTM) is a novel biophysical modeling‐based method for quantifying blood perfusion velocity. In this study, we examined regional changes in perfusion velocity throughout AD progression by combining QTM velocity measurements with Granger causality analysis using cross‐sectional data, as a secondary and exploratory analysis following our previous QTM work, aiming to offer a comprehensive view of the QTM velocity patterns based on the arterial territories.

**RESULTS:**

Reduced QTM velocity was observed in the middle cerebral artery (MCA)–supplied regions for patients with mild cognitive impairment. The MCA‐supplied temporal lobe is a driving region of QTM velocity changes in other brain regions.

**DISCUSSION:**

The temporal lobe supplied by the MCA is the earliest brain region to exhibit changes in QTM velocity, indicating its potential as an early biomarker for AD diagnosis.

**Highlights:**

- Quantitative transport mapping (QTM) velocity was significantly reduced in the middle cerebral artery (MCA)–supplied regions among patients with mild cognitive impairment, compared to cognitively normal individuals.
- The temporal lobe supplied by the MCA is a driving region of QTM velocity changes in other brain regions.
- The temporal lobe supplied by the MCA has great potential as an early biomarker for AD diagnosis.

## 1 BACKGROUND

Cerebral perfusion is markedly reduced in patients with Alzheimer's disease (AD).^1^ Constant cerebral perfusion is essential for maintaining normal brain function, ensuring the adequate delivery of oxygen, energy metabolites, and nutrients, while facilitating the removal of carbon dioxide and metabolic waste.^2^ Hence, investigating alterations in blood perfusion could provide valuable insights into the mechanism underlying the onset and progression of AD.

Arterial spin labeling (ASL) is a widely used non‐invasive technique for quantifying perfusion. Quantitative transport mapping (QTM), which fits multiple post‐labeling delay ASL (mPLD‐ASL) data to the transport equation, has been demonstrated to achieve greater accuracy in quantifying blood perfusion.^3^, ^4^ Our recent study revealed that QTM velocity can detect perfusion changes at the early stage of AD, with reduced QTM velocity observed in subjects with mild cognitive impairment (MCI) compared to normal controls (NCs) in both the cerebral cortex and the hippocampus.^4^ Therefore, QTM emerges as a promising approach for investigating dynamic perfusion changes associated with AD progression.

Most prior studies have reported reduced perfusion patterns in patients with AD, compared to NC.^4^, ^5^, ^6^ Several previous studies have demonstrated reduced cerebral blood flow (CBF) in subjects with MCI compared to NCs,^7^, ^8^, ^9^ while a meta‐analysis study^6^ and our recent study^4^ have shown that CBF does not show a significant difference in the cerebral cortex between those with MCI and NCs, suggesting that CBF is limited in capturing specific alterations in perfusion throughout the progression of AD. Given the relationships among perfusion alterations, arterial inflow, and blood supply, cerebral arterial territories were applied to segment the brain into vascular regions.^10^ This approach enabled the investigation of temporal alterations for regional QTM velocity throughout the progression of AD using Granger causality analysis (GCA).^11^, ^12^ The GCA method, applied to pseudo‐time‐series morphological data, has been used to explore the progression patterns of brain atrophy.^11^, ^13^, ^14^ This technique further facilitates the investigation of alteration in cerebral perfusion and enables the detection of perfusion pattern changes across disease progression.^12^

Amyloid beta (Aβ) deposition in the brain represents a key pathological hallmark associated with impaired glymphatic clearance in patients with AD. Animal studies have established that the glymphatic system accounts for 55% to 65% of Aβ protein clearance from the mouse brain.^15^ The glymphatic system facilitates fluid exchange between cerebrospinal fluid (CSF) in the perivascular space and interstitial fluid (ISF) within the brain's interstitial spaces,^16^ which has been linked to the pulsation of blood vessels.^17^ An animal study demonstrated that CBF is associated with brain clearance, underscoring the crucial roles of cardiovascular function and CBF in regulating the glymphatic system,^18^ as the blood flow, CSF, and ISF are key components of neurofluids.^19^ The diffusion tensor image (DTI) analysis along the perivascular space (ALPS) technique can estimate ISF diffusivity, which has been suggested to partially reflect glymphatic function.^20^ Recent studies have demonstrated that disruptions in perfusion and glymphatic dysfunction are associated with abnormalities in biomarkers of Aβ deposition in the brain.^21^, ^22^ Therefore, investigating the association between perfusion velocity and glymphatic function could enhance our understanding of the pathological process linking cardiovascular function and Aβ deposition in AD.

In our previous work, we found that QTM velocity in the cerebral cortex and hippocampus can distinguish those with MCI from NCs.^4^ In this study, we hypothesized that reductions in QTM velocity are progressively associated with the development of AD. As a secondary analysis of our previous work,^4^ we exploratorily applied GCA to regional QTM velocity data to evaluate the perfusion velocity patterns and the blood supply pathways using the arterial territories atlas throughout the progression of AD, identifying the earliest brain region exhibiting changes in QTM velocity and investigating arterial vessel impairment in AD.

## 2 METHODS

### 2.1 Subjects

This study was approved by the ethics committee of the Hainan General Hospital. All participants and/or their relatives were informed about this study and provided their written informed consent for data collection and sharing for research purposes.

**RESEARCH IN CONTEXT**

1. **Systematic review**: The authors reviewed the literature on cerebral perfusion in Alzheimer's disease (AD). Several studies have investigated cerebral perfusion in AD using cerebral blood flow (CBF), and reduced blood perfusion has been observed in patients with AD, while the patterns of blood perfusion changes, especially the changes of perfusion velocity pattern, across AD progression remain insufficiently explored.
2. **Interpretation**: Here, quantitative transport mapping (QTM) velocity, which has been reported to be sensitive to the CBF in detecting perfusion changes, was used to examine regional changes in perfusion velocity throughout AD progression. We found that there was reduced perfusion velocity in the middle cerebral artery (MCA)–supplied regions for patients with mild cognitive impairment. Moreover, the temporal lobe supplied by the MCA is a driving region of QTM velocity changes in other brain regions.
3. **Future directions**: These findings should be replicated in other large and diverse cohorts. The QTM velocity in the temporal lobe has the potential as an early biomarker for AD diagnosis. The vascular pathology of the MCA at the early stage of AD might also be worthy of investigation.

A total of 168 subjects, aged 55 to 90 years, were recruited from the community. All participants underwent neuropsychological tests and magnetic resonance imaging (MRI) examinations at Hainan General Hospital, Haikou, China. Exclusion included 12 participants who were unable to complete neuropsychological testing and 6 participants who could not remain still during the MRI or had severe image artifacts, leaving 150 eligible subjects. Probable AD was diagnosed based on the criteria set by the National Institute on Aging and Alzheimer's Association (NIA‐AA),^23^ while MCI was diagnosed according to the Petersen criteria.^24^ NC was defined based on clinical interviews, corroborated by a Mini‐Mental State Examination (MMSE) score > 26 and a Clinical Dementia Rating (CDR) score of 0.^25^

Note that 128 subjects in this study are from the same cohort as in our prior publication,^4^ in which we evaluate the diagnostic group difference in both QTM velocity and CBF. We continuously collected older volunteers and added 22 more subjects to this work. In this work, we aim to investigate the QTM velocity pattern and identify the corresponding blood supply pathways throughout the progression of AD cross‐sectionally.

### 2.2 Neuropsychological tests and cognitive outcomes

To assess cognitive status, four neuropsychological tests were administered.^26^

The MMSE is a 30‐item screening tool used to summarize cognitive abilities, including orientation, memory, attention, and language.^27^ We used the total score in our analysis.

The Trail‐Making Test Parts A (TMT)‐A and B (TMT‐B) require participants to draw lines connecting circles containing numbers (A) or alternating letters and numbers (B) in ascending order.^28^ The time needed to complete each test serves as an indicator of processing speed and executive function.

In the Rey Auditory Verbal Learning Test (RAVLT), a list of 15 words is read five times. The participant is asked to recall the words after each presentation (immediate recall and learning). After a 20 minute delay, the participant is asked to recall the words again (delayed recall). We used the mean number of words recalled for the first three trials (immediate recall scores) as indicators of episodic memory and analyzed the total number of words recalled after the 20 minute delay (delayed recall score).^29^

In the semantic verbal fluency test (VFT), participants are asked to name as many animals as possible within 60 seconds. The total number of animals named was used as an indicator of semantic fluency.^30^

### 2.3 MRI data acquisition

All participants underwent MR examinations using a 3.0T MR scanner (Prisma, Siemens) with a 64‐channel head/neck receiver coil. The imaging protocol included a three‐dimensional (3D) magnetization‐prepared rapid acquisition gradient‐echo (MPRAGE T1W) sequence for anatomical imaging, a 3D pseudo‐continuous arterial spin labeling (PCASL) sequence with five post‐labeling delay (PLD) times for perfusion quantification,^31^, ^32^ and a multiband echo‐planar imaging sequence for DTI. Scanning parameters were as follows: (1) MPRAGE T1W: echo time (TE) = 2.26 ms; repetition time (TR) = 2300 ms; inversion time = 900 ms; flip angle = 8°; slice thickness = 1 mm; field of view (FOV) = 256 × 256 mm^2^; voxel size = 1 × 1 × 1 mm^3^; (2) PCASL: TE = 37.78 ms; TR = 4200 ms; PLD = 500, 1000, 1500, 2000, 2500 ms; slice thickness = 3 mm; FOV = 240 × 240 mm^2^; voxel size = 2.5 × 2.5 × 3 mm^3^; (3) DTI: TE = 65 ms; TR = 4500 ms; 64 gradient directions for b = 1000 s/mm^2^; 12 images for b = 0 s/mm^2^; slice thickness = 2 mm; FOV = 224 × 224 mm^2^; voxel size = 2 × 2 × 2 mm^3^. Routine MR sequences (T2W and T2‐fluid‐attenuated inversion recovery) were also included to detect brain abnormalities.

### 2.4 QTM reconstruction

We reconstructed QTM from the mPLD‐ASL data using the mass conservation equation:^3^, ^4^

$$\partial_{t}c\;\left(r,t\right)=-\nabla \cdot \left(c\left(r,t\right)u\left(r\right)\right)+\nabla \cdot \left(D\left(r\right)\nabla c\left(r,t\right)\right)-\lambda c\left(r,t\right),$$

where c(r,t) represents the tracer concentration at the location r and time t, u(r) denotes the time‐invariant voxel‐wise average tracer velocity, D(r) is the apparent diffusion coefficient, and λ is the signal decaying rate. For MR‐labeled endogenous water molecules in ASL data, the λ=1/T1b, and T1b=1.65s is the longitudinal relaxation time (*T*1) of blood.^33^ For perfusion estimation, D(r) can be considered ignorable, as the diffusion process occurs at a much slower rate than blood perfusion. The reconstruction of QTM velocity is then performed using the optimization approach described below:^4^, ^34^

$$u\;=\mathrm{argmi}n_{u}\sum_{t=1}^{N_{t}-1}∥\partial_{t}c+\nabla \cdot \left(cu\right)+\lambda c{∥}_{2}^{2}+\alpha ∥\nabla u{∥}_{1},$$

where α is the regularization parameter in the optimization to enforce a region‐wise smooth solution. The reconstruction processing of velocity u was performed using in‐house code implemented in MATLAB. The magnitude of u denoted as ∥u∥ in the L2 norm to represent the QTM velocity magnitude. The velocity u is derived from the inverse problem of the mass conservation equation, which needs to be physiologically validated in the brain, although the model was numerically validated in the kidney.^3^ Theoretically, the QTM velocity ∥u∥ is a spatial‐temporal average of velocity across all the arterial vessels within the voxel (a typical voxel size in ASL is an ≈ 2.5 mm isotropic cube, which contains numerous microscale vessels) and the data acquisition duration;^35^ therefore, we are safe to consider the model derived ∥u∥ as a relative perfusion velocity before it is physiologically or cross‐modality validated. The magnitude of QTM velocity ∥u∥ (≈ 10 mm/s) is out of the range of blood velocity in capillaries (≈ 1 mm/s) or arteries (up to 800 mm/s), which might be due to the spatiotemporal nature of ∥u∥ derived from the QTM model. Hence, in this work, we treat QTM velocity as a relative measure of perfusion velocity and omit its unit.

### 2.5 QTM normalization and segmentation

QTM was warped into the Montreal Neurological Institute (MNI) space as follows: (1) the QTM velocity magnitude images were registered to the structural T1W images; (2) the structural images were warped to the standard MNI brain template, segmented into probability maps of gray matter (GM), white matter, and CSF, and the transformation matrix was saved; (3) the saved transformation matrix derived from the structural images was applied to the QTM images.

In MNI space, cerebral arterial territories were used as an atlas and categorized into 26 cerebral regions,^10^ and the abbreviations of the regions and their corresponding Anatomical Automatic Labeling (AAL) brain regions are provided in Table S1 in supporting information. The arterial atlas is available at https://www.nitrc.org/projects/arterialatlas10.

### 2.6 The GCA of regional QTM velocity

**FIGURE 1:**
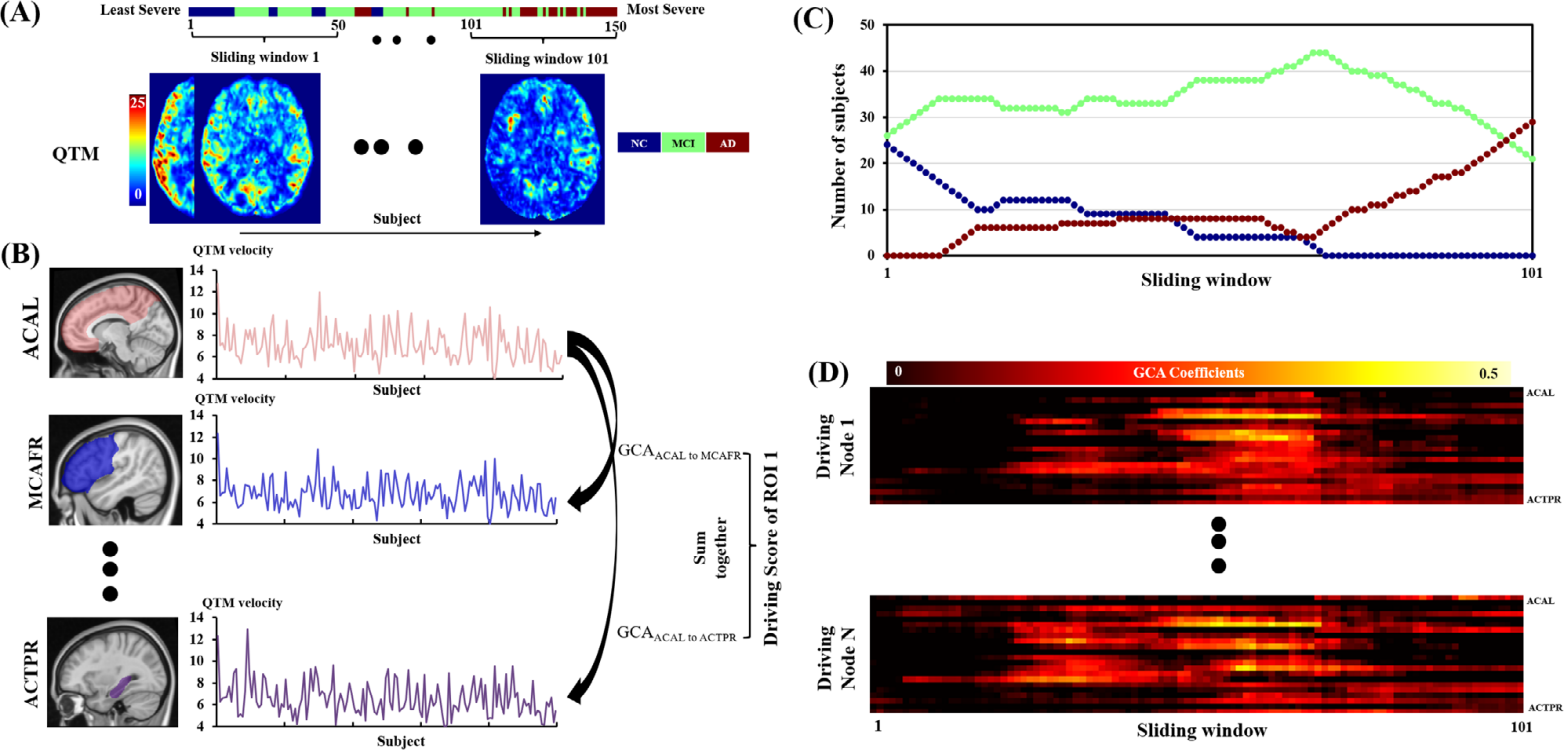
GCA of regional QTM velocity for the whole brain. A, Construction of pseudo‐time series of QTM velocity according to cognitive order from high to low, and illustration of the GCA sliding window with a window size of 50. B, The GCA coefficients were calculated between regions according to pseudo‐time series of QTM velocity. C, The distribution of the number of subjects for each sliding window. D, The sliding‐window GCA coefficients for driving regions. ACAL, anterior cerebral artery left; ACTPR, anterior choroidal and thalamoperfurators right; GCA, Granger causality analysis; MCAFR, frontal pars of middle cerebral artery right; QTM, quantitative transport mapping; ROI, region of interest.

Our hypothesis is that the disease progression of multiple time points for one patient has a similar trajectory to the progression in populations. The GCA method was applied to QTM velocity maps identifying the pattern of regional QTM velocity changes across AD progression. Specifically, the quantification of AD progression for all subjects was ranked from highest to lowest MMSE score.^36^ For subjects with identical MMSE scores, they were ranked from highest to lowest based on immediate recall scores from the RAVLT test, as shown in Figure 1A. This ranked order of subjects is intended to represent the progression of AD onset and development using cross‐sectional data. The corresponding final order of QTM velocity served as a “pseudo‐time series” and was used in all the following analyses to identify the earliest region of QTM velocity change. Given two “pseudo‐time series” $\mathrm{QT}M_{i}$ and $\mathrm{QT}M_{j}$ velocities for the *i*‐th and *j*‐th regions, a first‐order GCA model describing the relationship of QTM velocity impact between region *i* and region *j* can be modeled as follows:^11^

$${\;\mathrm{QTM}}_{j}^{t}=A_{ij}{\;*\mathrm{QTM}}_{i}^{t-1}+B_{j}{*\mathrm{QTM}}_{j}^{t-1}+\varepsilon_{t},\;\mathrm{for}\;\mathrm{all}\;j\neq i\;and\;j\left[1,\;26\right].$$

The t represents the pseudo‐time point corresponding to the subject order based on cognitive scores and ranges from 2 to the total number of subjects, *N*. The GCA coefficient $\;A_{ij}$ represents the effect of QTM velocity at the pseudo‐time point t−1 in region i to predict QTM velocity at the pseudo‐time point t in region j, and the coefficient $B_{j}$ describes the effect of QTM velocity at the pseudo‐time point t−1 to predict QTM velocity at the pseudo‐time point t for region j itself; the $\varepsilon_{t}$ represents the model error. A positive GCA coefficient $A_{ij}$ indicates that the QTM velocity in the region j changes in the same direction as those in the region i, whereas a negative GCA coefficient $A_{ij}$ indicates an opposite impact of QTM velocity in the region i on that in region j.

### 2.7 The driving regions derived from the GCA coefficient

To extract the driving regions with the most important driving effect on QTM velocity alterations, we calculated the driving score for 22 regions where QTM velocity showed significant differences among AD, MCI, and NC groups (see Section 3 for details). The driving score for the region i, $A_{i}$ is defined as the sum of the GCA coefficients $A_{ij}$ from region i to all other regions $j:\;\;A_{i}=\sum_{j\neq i}^{N}A_{ij}$, as shown in Figure 1B. Positive and negative driving scores were calculated separately as the sum of all positive and negative GCA coefficients j, respectively $A_{i}^{pos}=\sum_{j\neq i}^{N}A_{ij}\;if\;A_{ij}\geq 0\;\mathrm{and}A_{i}^{neg}=\sum_{j\neq ii}^{N}A_{ij}\;if\;A_{ij}\leq 0.$ Regions with significant driving scores, as determined by permutation‐based tests (see details in Section 2.10), were defined as the driving regions during AD progression, ordered by cognition. Therefore, the driving region is defined as a region that has QTM velocity changes prior to other regions.

### 2.8 Sliding‐window GCA for the driving regions

To investigate the driving effect of QTM velocity alterations for the driving regions at various stages of AD, a sliding‐window GCA method was performed. This method applied a sliding window on pseudo‐time‐series QTM velocity maps and calculated GCA coefficients from driving regions to all other regions on each sliding window. As shown in Figure 1A, the pseudo‐time‐series QTM velocity maps were ranked by cognitive function from high to low, as mentioned above. According to a previous study,^11^ the sliding‐window length was set to 50 subjects: the 1st window included the 1st subject to the 50th, the second window included the subject from the 2nd to the 51st, and so on to the last window, which consisted of the 101st to 150th subjects. As shown in Figure 1C, there were fewer AD subjects in the windows corresponding to the least severe cases, in which subjects have better cognition, and fewer NC subjects in windows representing more severe conditions. Because of the large number of MCI subjects in this study, the distribution of MCI across each sliding window was relatively uniform. For each sliding window, given N brain regions, there were *N* – 1 GCA coefficients calculated for each driving region. The sliding window GCA coefficient maps are shown in Figure 1D.

### 2.9 ALPS index calculation

To estimate ISF dynamics, the ALPS index was used^22^ and was defined as follows:

$$\mathrm{ALPS}\;\mathrm{index}=\frac{\mathrm{mean}\left(D_{xproj},\;D_{xassoc}\right)}{\mathrm{mean}\left(D_{yproj},\;D_{zassoc}\right)}$$

where *D_xproj_* and *D_yproj_* represent *x* axis and *y* axis diffusivity in regions of projection fibers, while *D_xassoc_* and *D_zassoc_* represent *x* axis and *z* axis diffusivity in regions of association fibers. Diffusion metric images were generated using DSI Studio software (https://dsi‐studio.labsolver.org/). To avoid bias related to manually drawn regions of interest, an atlas‐based approach was used in this study.^37^ Briefly, each participant's fractional anisotropy (FA) map was coregistered to the FA map template of the ICBM‐DTI‐81 atlas^38^ using non‐linear registration in SPM12.^39^ Other diffusion metric maps of each participant were warped using the registration transform matrix derived from the FA map. The ALPS index was automatically computed according to the above formula for both the left and right hemispheres, and the mean ALPS index of both sides was reported.^40^

### 2.10 Statistical analysis

To test the significance of driving scores in GCA, a permutation‐based test was applied.^41^ For each permutation, the order of the subjects was randomized, and the GCA was conducted for this newly randomized pseudo‐time series, with all the GCA coefficients recorded. After 1000 permutations, driving scores were calculated, resulting in *N* × 1000 positive and *N* × 1000 negative driving scores for the brain regions. We defined scores within the 5% tails of the distribution as significant at *p *< 0.05. The regions that had significant driving scores were defined as the driving regions during AD progression. Similarly, the significance of the sliding‐window GCA coefficients was tested using another 1000 permutation tests. For each permutation, the 50 subjects within the sliding window were randomly ordered, and the GCA coefficients were calculated. QTM velocity values were compared among diagnostic groups using a one‐way analysis of covariance with age, sex, and years of education as covariates. Post hoc multiple Bonferroni comparisons were performed to evaluate statistical differences between diagnostic groups. The association between cognitive scores and QTM velocity measures was assessed using Pearson or Spearman correlation analysis. Finally, linear regression analysis was applied to investigate the association between QTM velocity and the ALPS index, adjusting for age, sex, and education. Analysis of covariance (ANCOVA) was used to compare if the associations between QTM velocity and ALPS were significant across the diagnostic groups. Note that all *r* values reported represent the correlation coefficients, and all *p* values reported are false discovery rate adjusted for multiple comparisons. All significance tests were two sided with *α *= 0.05 set as the significance threshold.

## 3 RESULTS

### 3.1 Subject information and demographics

Among 150 eligible subjects, 37 were diagnosed with probable AD, 85 with MCI, and 28 as NC. There was no significant difference in sex among the three groups (*p* = 0.227). The mean age was significantly higher in the AD group compared to the MCI group (*p* = 0.005), and years of education were significantly higher in the NC group compared to both the MCI (*p* < 0.001) and AD (*p* < 0.001) groups. Cognition scores, including MMSE, immediate recall scores, delayed recall scores, TMT‐A, TMT‐B, and VFT, were significantly different among the three groups (all *p* < 0.001). The results are summarized in Table 1.

**TABLE 1:** Demographics and clinical characteristics of the study population.

	NC (*n *= 28)	MCI (*n *= 85)	Probable AD (*n *= 37)	*F*/*χ*2 value	*p*value
**Age**	69.61 ± 6.65	67.56 ± 5.82	71.78 ± 8.41	6.268	0.006^a^
**Sex F/M**	17/11	48/37	27/10	2.966	0.227^b^
**Education level (years)**	13.68 ± 3.95	10.10 ± 3.81	9.58 ± 3.95	9.797	<0.001^a^
**MMSE**	28.54 ± 1.55	24.80 ± 3.80	16.57 ± 7.40	59.596	<0.001^a^
**Immediate recall score**	6.37 ± 1.69	4.23 ± 1.63	2.86 ± 1.94	32.992	<0.001^a^
**Delayed recall score**	6.14 ± 2.01	3.40 ± 2.44	2.19 ± 2.61	21.418	<0.001^a^
**TMT‐A**	65.36 ± 25.21	94.59 ± 44.05	167.03 ± 79.96	32.690	<0.001^a^
**TMT‐B**	148.07 ± 42.52	199.87 ± 55.12	274.05 ± 35.33	35.847	<0.001^a^
**VFT**	19.93 ± 4.07	13.95 ± 5.13	9.03 ± 4.23	40.998	<0.001^a^

Abbreviations: AD, Alzheimer's disease; MCI, mild cognitive impairment; MMSE, Mini‐Mental State Examination; NC, normal cognition; TMT‐A, Trail‐Making Test Part A; TMT‐B, Trail‐Making Test Part B; VFT, verbal fluency test.

^a^ One‐way analysis of variance;

^b^χ^2^ test.

### 3.2 GCA coefficients and driving scores

**FIGURE 2:**
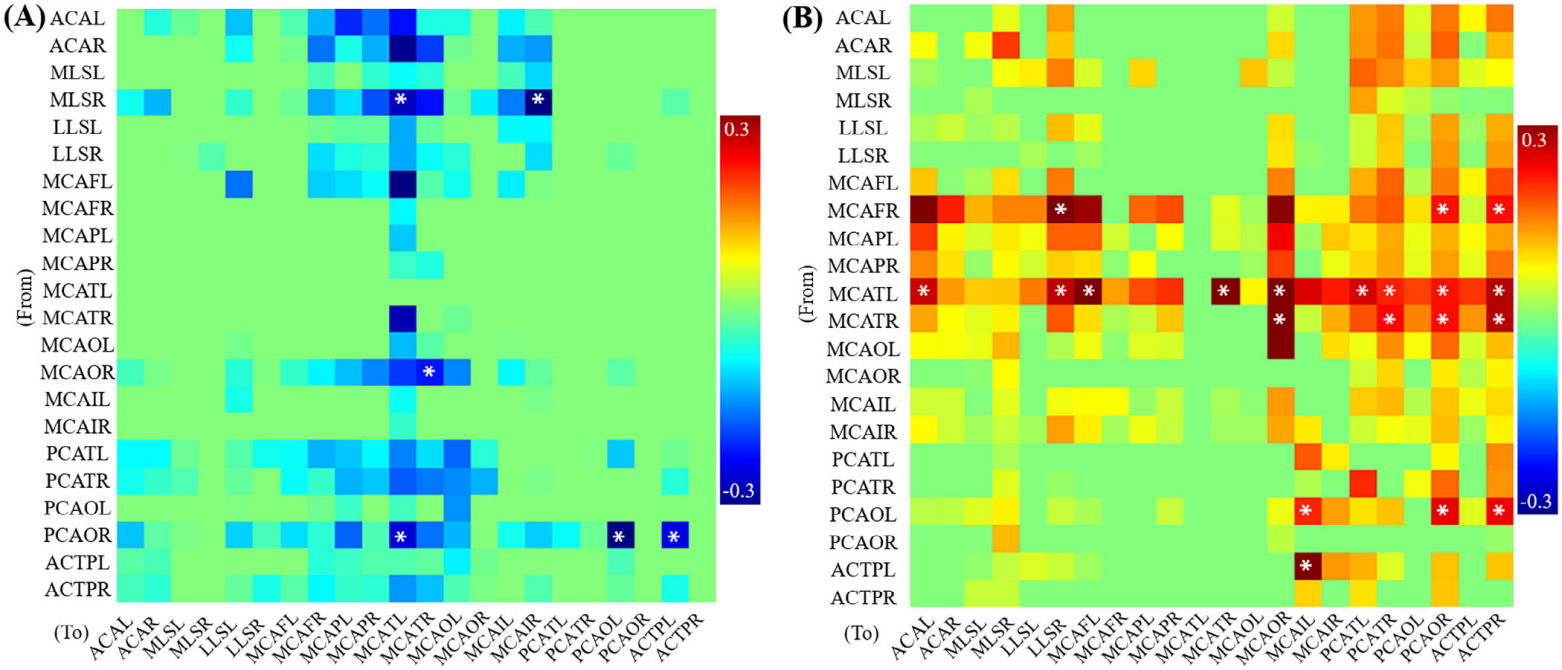
Maps of negative (A) and positive GCA coefficients (B) for each pair of brain regions show three significantly negative driving regions and five significantly positive driving regions. A negative GCA coefficient indicates that the QTM velocity changes in the opposite direction between the pair regions, and a positive GCA coefficient indicates that the QTM velocity changes occur in the same direction. *Indicates that the GCA coefficient was significant. ACAL, anterior cerebral artery left; ACAR, anterior cerebral artery right; ACTPL, anterior choroidal and thalamoperforators left; ACTPR, anterior choroidal and thalamoperforators right; GCA, Granger causality analysis; LLSL, lateral lenticulostriate left; LLSR, lateral lenticulostriate right; MCAFL, frontal pars of middle cerebral artery left; MCAFR, frontal pars of middle cerebral artery right; MCAIL, insular pars of middle cerebral artery left; MCAIR, insular pars of middle cerebral artery right; MCAOL, occipital pars of middle cerebral artery left; MCAOR, occipital pars of middle cerebral artery right; MCAPL, parietal pars of middle cerebral artery left; MCAPR, parietal pars of middle cerebral artery right; MCATL, temporal pars of middle cerebral artery left; MCATR, temporal pars of middle cerebral artery right; MLSL, medial lenticulostriate left; MLSR, medial lenticulostriate right; PCAOL, occipital pars of posterior cerebral artery left; PCAOR, occipital pars of posterior cerebral artery right; PCATL, temporal pars of posterior cerebral artery left; PCATR, temporal pars of posterior cerebral artery right; QTM, quantitative transport mapping.

QTM velocity exhibited significant differences among the AD, MCI, and NC groups across 22 regions in the brain MRI arterial territories atlas, which were subsequently applied for GCA. These 22 significant regions include 12 middle cerebral artery (MCA) regions, 4 anterior cerebral artery (ACA) regions, and 6 posterior cerebral artery (PCA) regions. The GCA coefficients for each pair of brain regions are shown in Figure 2. Six negative regional GCA coefficients, marked with asterisks in Figure 2A, were identified as significant, with the driving regions being medial lenticulostriate right (MLSR), occipital pars of middle cerebral artery right (MCAOR), and occipital pars of posterior cerebral artery right (PCAOR). In contrast, most GCA coefficients exhibited significantly positive values, as indicated by asterisks in Figure 2B. The five positive driving regions included frontal pars of middle cerebral artery right (MCAFR), the temporal pars of middle cerebral artery left (MCATL), the temporal pars of middle cerebral artery right (MCATR), the occipital pars of the posterior cerebral artery left (PCAOL), and the anterior choroidal and thalamoperforators left (ACTPL).

**FIGURE 3:**
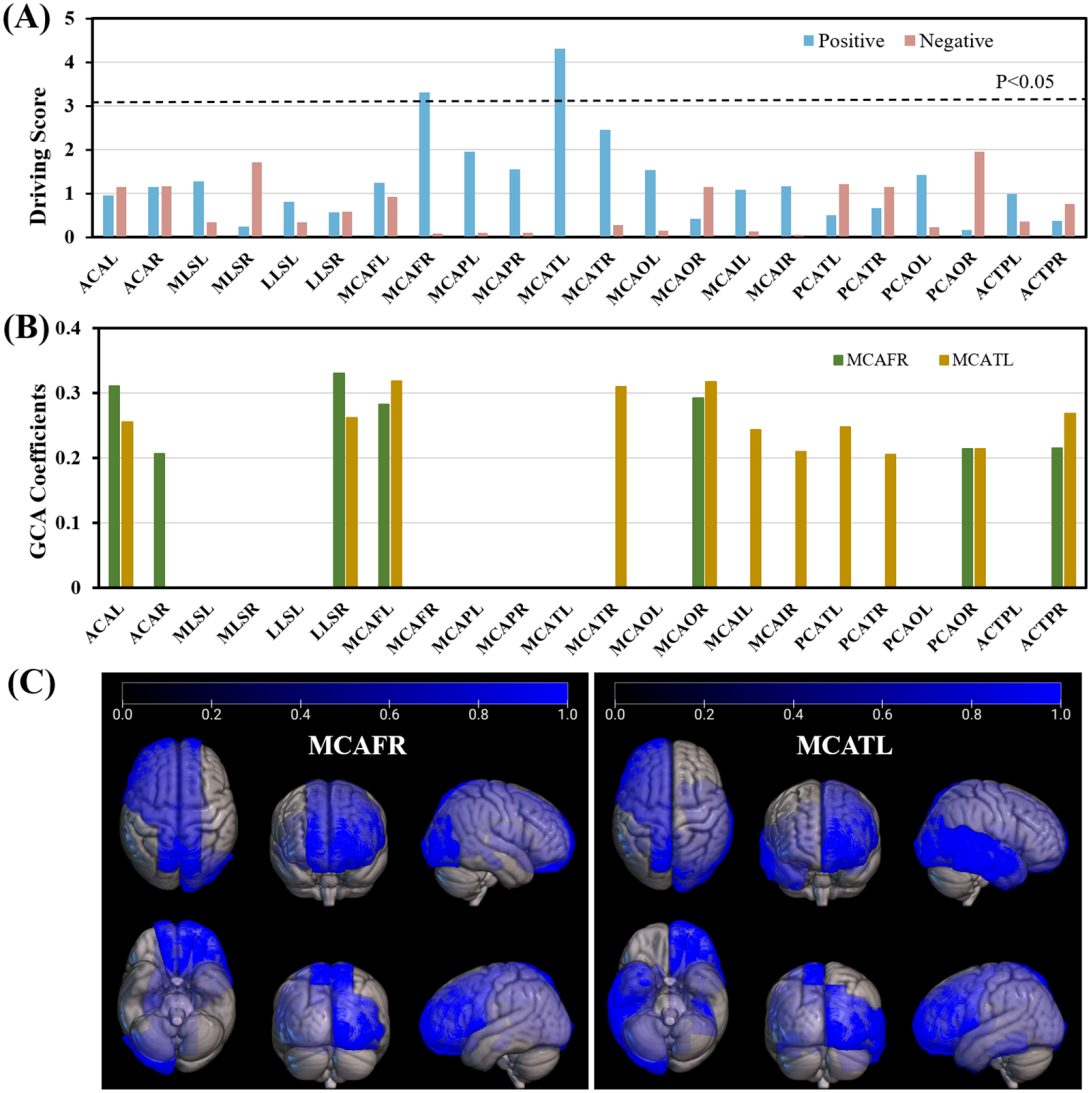
Driving scores reveal two driving regions (MCAFR and MCATL) with significantly positive driving scores. A, Driving scores for 22 brain regions exhibiting significant group differences among AD, MCI, and NC. Two brain regions, MCAFR and MCATL, with significant positive driving scores were identified as driving regions (permutation test, *p *< 0.05). B, GCA coefficients from the driving region (MCAFR or MCATL) to all other brain regions. C, Brain regions with significant GCA coefficients derived from MCAFR (left) and MCATL (right). ACAL, anterior cerebral artery left; ACAR, anterior cerebral artery right; ACTPL, anterior choroidal and thalamoperforators left; ACTPR, anterior choroidal and thalamoperforators right; AD, Alzheimer's disease; GCA, Granger causality analysis; LLSL, lateral lenticulostriate left; LLSR, lateral lenticulostriate right; MCAFL, frontal pars of middle cerebral artery left; MCAFR, frontal pars of middle cerebral artery right; MCAIL, insular pars of middle cerebral artery left; MCAIR, insular pars of middle cerebral artery right; MCAOL, occipital pars of middle cerebral artery left; MCAOR, occipital pars of middle cerebral artery right; MCAPL, parietal pars of middle cerebral artery left; MCAPR, parietal pars of middle cerebral artery right; MCATL, temporal pars of middle cerebral artery left; MCATR, temporal pars of middle cerebral artery right; MCI, mild cognitive impairment; MLSL, medial lenticulostriate left; MLSR, medial lenticulostriate right; NC, normal cognition; PCAOL, occipital pars of posterior cerebral artery left; PCAOR, occipital pars of posterior cerebral artery right; PCATL, temporal pars of posterior cerebral artery left; PCATR, temporal pars of posterior cerebral artery right.

As shown in Figure 3A, two regions, MCAFR and MCATL, showed significantly positive driving scores (permutation test, *p* < 0.05), while none of the negative driving scores reached a significant level. The MCAFR region includes the right frontal lobe and anterior cingulate, and the MCATL region includes the left temporal lobe, hippocampus, parahippocampal gyrus, and amygdala. As shown in Figures 3B and 3C, seven regions had GCA coefficients > 0.2 for the driving region of MCAFR, and 11 regions had GCA coefficients > 0.2 for the driving region of MCATL, indicating that QTM velocity reduction in both driving regions preceded QTM velocity reduction in other regions.

### 3.3 Group difference of QTM velocity

**FIGURE 4:**
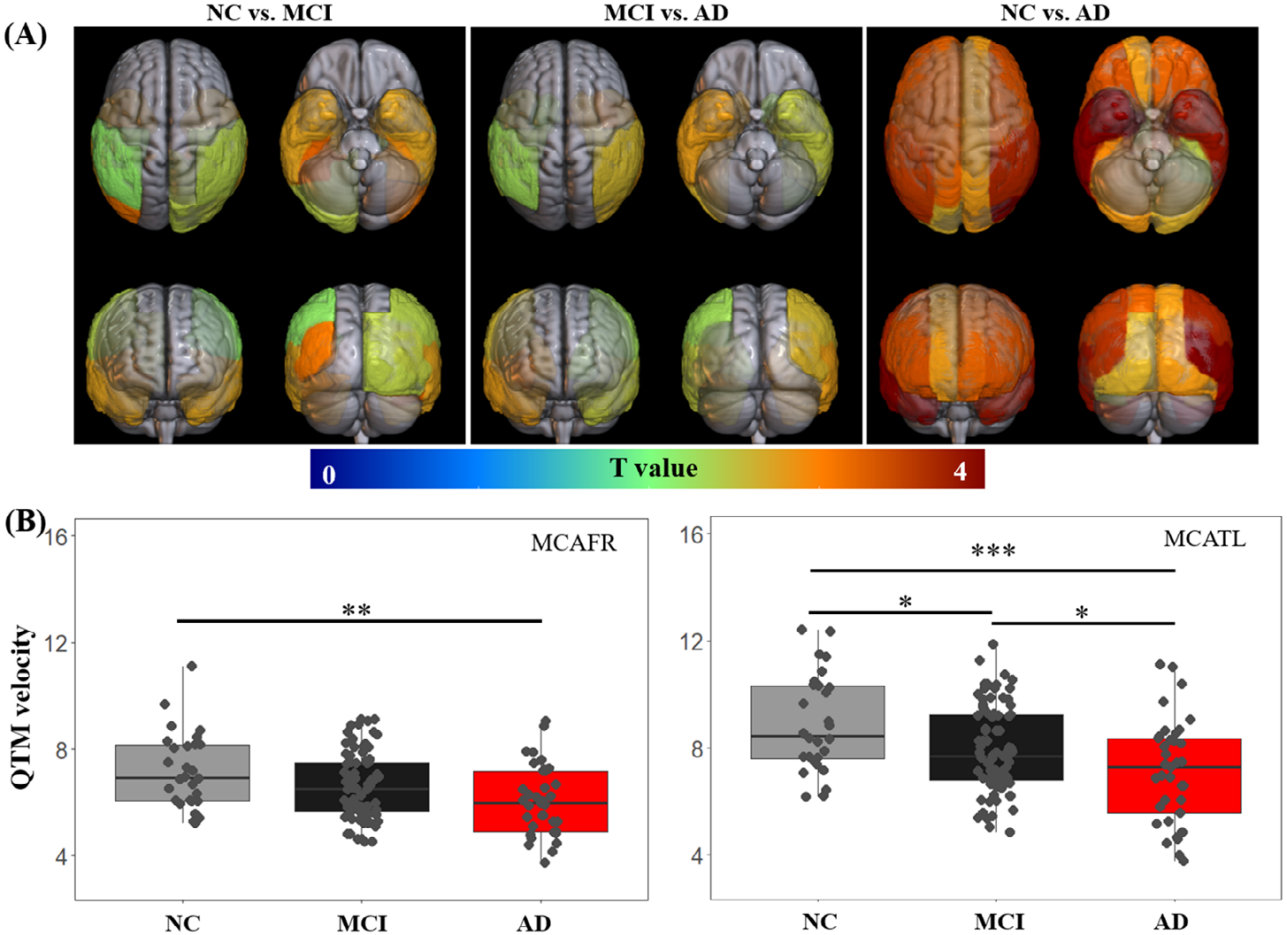
Group differences in QTM velocity across various cerebrum regions. A, *T* value distribution from *t* test comparing QTM velocity across diagnosis groups for all brain regions. B, Box plot illustrating QTM velocity differences across groups for the two driving regions. The colored regions in (A) indicate significant differences (*p *< 0.05 with false discovery rate correction). *p < 0.05, **p < 0.01, and *** *p *< 0.001. AD, Alzheimer's disease; MCAFR, frontal pars of middle cerebral artery right, MCATL, temporal pars of middle cerebral artery left; MCI, mild cognitive impairment; NC, normal cognition; QTM, quantitative transport mapping.

QTM velocity showed significant differences across 22 brain regions among diagnostic groups, with the *T* value of significant comparisons for QTM velocity in the whole brain displayed in Figure 4A. Significant hypoperfusion was observed in MCI patients in the MCA‐supplied brain regions (MCATL, MCATR, MCAOL, and MCAOR) and the PCA‐supplied brain regions (PCATR, PCAOL, and PCAOR). In contrast, no significant differences in QTM velocity were observed in ACA‐ and vertebrobasilar artery (VB)‐supplied territories.

Figure 4B shows the QTM velocity difference among diagnostic groups for the two driving regions, MCAFR and MCATL. In MCAFR, QTM velocity was significantly reduced in the AD group compared to the NC group (*p* = 0.002), while only marginal differences were observed between NC and MCI groups (*p* = 0.095) and between MCI and AD groups (*p* = 0.106). These observations suggested that QTM velocity change in MCAFR drove its changes in other regions only in the late stage of the AD continuum. In MCATL, QTM velocity was significantly reduced in the AD group compared to the MCI group (*p* = 0.048) and the NC group (*p* < 0.001). Furthermore, QTM velocity was reduced in MCI patients compared to NC (*p* = 0.029), suggesting that the MCATL territory could represent an early region of blood perfusion velocity change transition from NC to MCI. The group differences of the QTM velocity in the subregions of MCATL corresponding to the AAL atlas brain regions are shown in Table S2 in supporting information. These findings suggest that changes in blood perfusion velocity in the MCATL may occur earlier than those in the MCAFR during the onset and progression of AD.

### 3.4 Sliding‐window GCA of the driving regions

**FIGURE 5:**
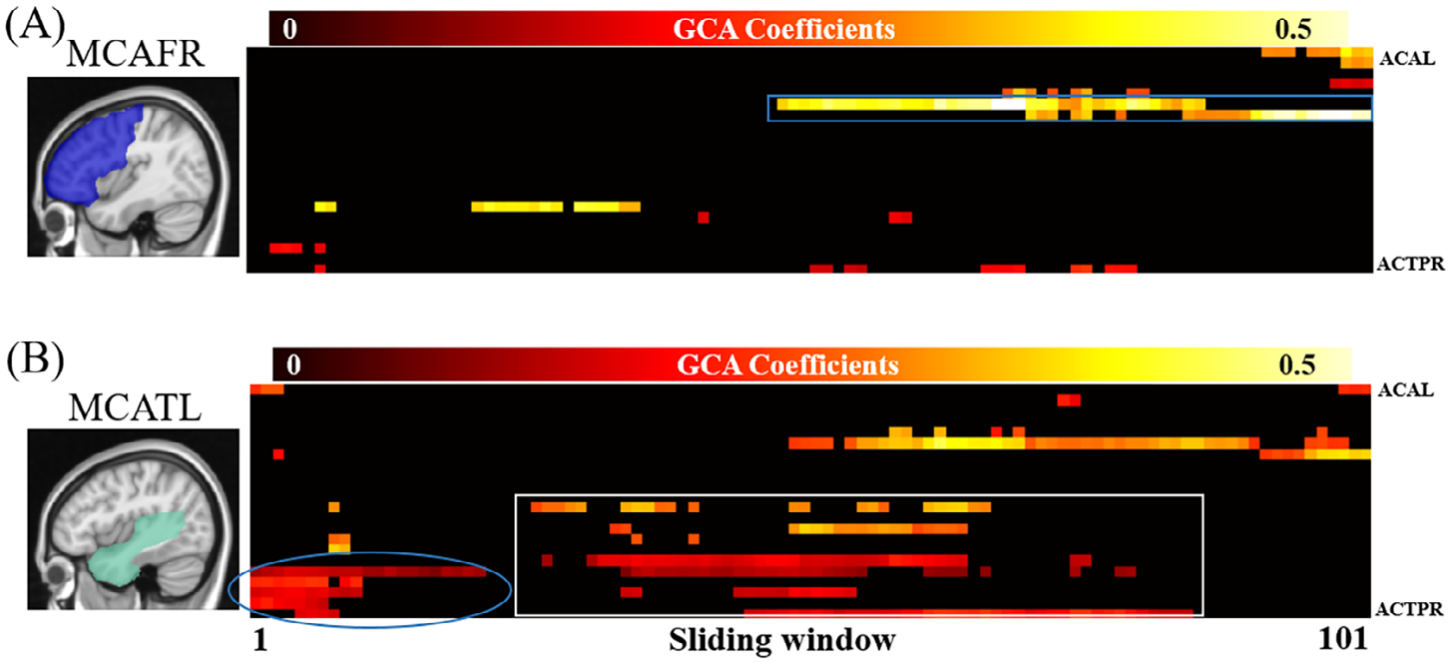
Sliding window GCA coefficients for the two driving regions exhibit dynamic changes. Sliding window GCA coefficients from (A) MCAFR and (B) MCATL to all other regions are displayed. Only significant GCA coefficients (determined by permutation test, *p *< 0.05) are highlighted in color. These results show that the change of perfusion velocity in MCA supplied temporal lobe region drives the change of perfusion velocity in other regions at very early stage (blue oval shape highlighted) of cognitive alteration. ACAL, anterior cerebral artery left; ACTPR, anterior choroidal and thalamoperforators right; GCA, Granger causality analysis; MCAFR, frontal pars of middle cerebral artery right; MCATL, temporal pars of middle cerebral artery left.

In Figure 5, as the window slid from the least to the most severe subjects, the GCA coefficients from the two driving regions to other regions exhibited dynamic changes. The significant GCA coefficients were sparse across all windows for MCAFR (Figure 5A) but were denser in the less and moderately severe windows for MCATL (Figure 5B). Moreover, the GCA coefficients for both driving regions were higher in the moderately and most severe windows compared to the less severe windows. For lateral lenticulostriate right (LLSR) and MCAFL, both driving regions exhibited similar patterns, with significant GCA coefficients observed during the later stage of AD (MMSE score ≤ 24, blue box in Figure 5A). For MCATL, significant GCA coefficients were identified in several regions, including PCATR, PCAOL, PCAOR, ACTPL, and ACTPR, during the early stage of AD (MMSE score ≥ 26, blue ellipsoid in Figure 5B), suggesting that QTM velocity alteration may precede cognition changes. QTM velocity alterations in MCATL precede QTM velocity changes in several regions (MCATR, MCAOR, PCATL, PCATR, PCAOR, and ACTPR) during the middle stage of MCI (white box in Figure 5B).

### 3.5 QTM velocity of driving regions linked with cognitive function

**FIGURE 6:**
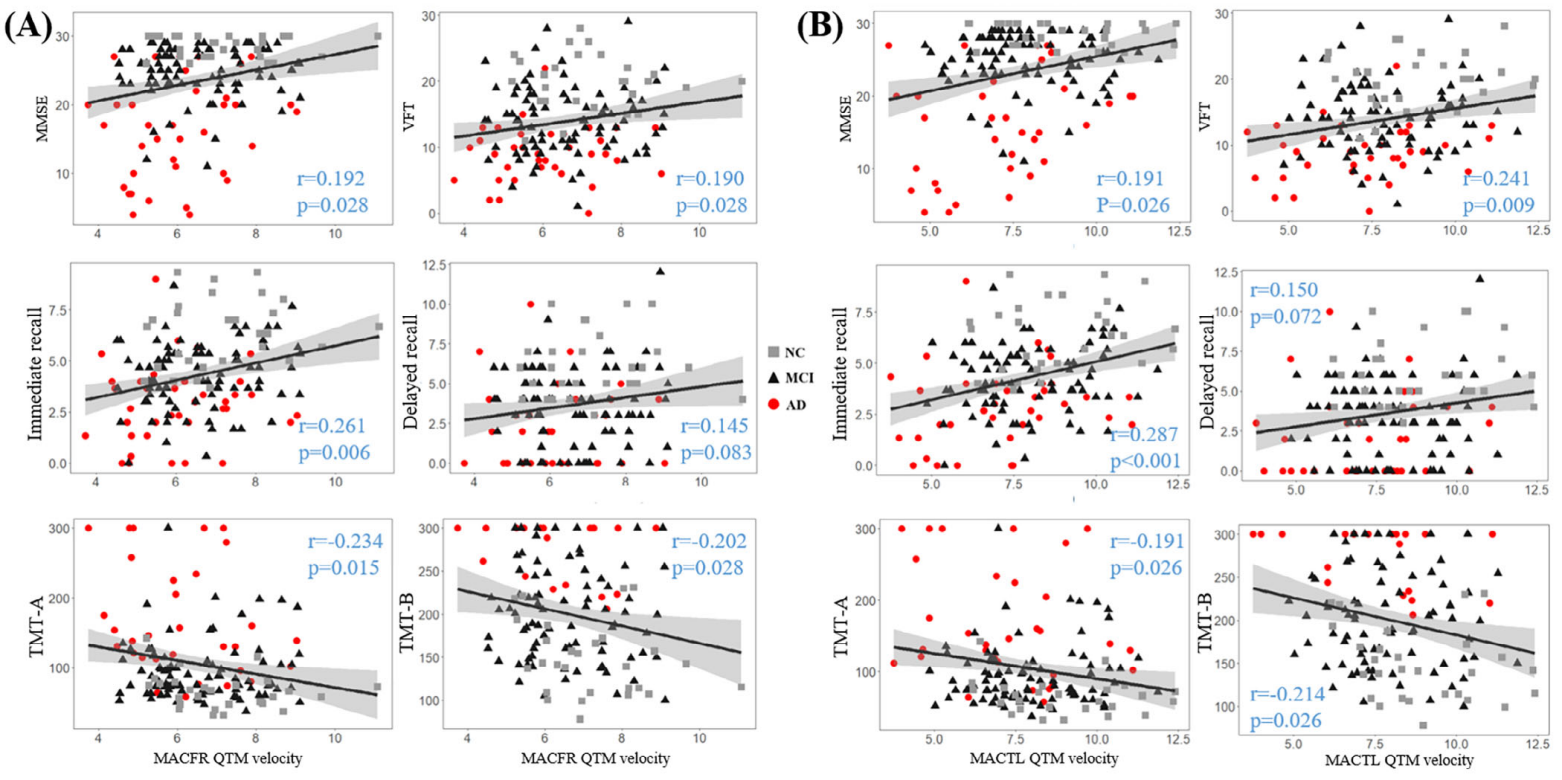
QTM velocity in the driving regions in relation to cognitive measures for all subjects. A, QTM velocity in MCAFR in relation to MMSE, VFT, immediate recall, delayed recall, TMT‐A, and TMT‐B. B, QTM velocity in MCATL in relation to the same cognitive measures. Each column uses the same *x* axes. All *p* values were adjusted using false discovery rate correction. MCAFR, frontal pars of middle cerebral artery right; MCATL, temporal pars of middle cerebral artery left; MMSE, Mini‐Mental State Examination; QTM, quantitative transport mapping; TMT‐A, Trail‐Making Test Part A; TMT‐B, Trail‐Making Test Part B; VFT, verbal fluency test.

Correlation analysis was performed to assess the relationship between cognitive abilities and QTM velocity in both driving regions, as shown in Figure 6. Figure 6A shows the relationship between QTM velocity in MCAFR and cognitive measures. Similarly, Figure 6B shows the relationship for the MCATL region. In the MCAFR region, QTM velocity showed positive correlations with MMSE (*r* = 0.192, *p* = 0.028), VFT (*r* = 0.190, *p* = 0.028), and immediate recall (*r* = 0.261, *p* = 0.006), while negative correlations with TMT‐A (*r* = −0.234, *p* = 0.015) and TMT‐B (*r* = −0.202, *p* = 0.028). Similar correlations were identified between QTM velocity and cognitive measures in the MCATL region.

### 3.6 Association between QTM velocity and DTI‐ALPS index

To evaluate the association between the perfusion velocity and ISF dynamics, as measured by DTI‐ALPS and potentially reflecting glymphatic function, we performed a linear regression analysis, adjusting for age, sex, and education. Table 2 presents the association coefficients between QTM velocity in 12 MCA territories and the ALPS index for the whole cohort and subgroups (NC, MCI, and AD groups). In the whole cohort, positive associations were observed between QTM velocity and the ALPS index in 14 brain blood–supplied territories, including ACAL, MLSL, MLSR, lateral lenticulostriate left (LLSL), MCAFL, MCAFR, MCAPL, MCAPR, MCATL, MCATR, MCAOL, MCAOR, insular pars of middle cerebral artery left (MCAIL), and insular pars of middle cerebral artery right (MCAIR). No significant associations were found between QTM velocity and the ALPS index in the NC, MCI, or AD groups when assessed separately, and there is also no diagnostic group difference for the associations between QTM velocity and ALPS based on ANCOVA. These findings suggest that ISF diffusivity may be partially driven by blood perfusion, highlighting a potential association among blood perfusion velocity, cardiovascular function, and glymphatic function.^18^

**TABLE 2:** Association between QTM velocity and ALPS index in 12 MCA territories for the whole cohort, NC, MCI, and probable AD groups.

brain region	Whole cohort (*n* = 150)		NC (*n* = 28)		MCI (*n* = 85)		Probable AD (*n* = 37)	
	*r*	*p*	*r*	*p*	*r*	*p*	*r*	*p*
**LLSL**	**0.266**	0.007	0.331	0.920	0.204	0.208	0.271	0.472
**LLSR**	0.125	0.249	−0.094	0.920	0.065	0.738	0.163	0.504
**MCAFL**	**0.239**	0.014	0.168	0.920	0.210	0.205	0.242	0.472
**MCAFR**	**0.215**	0.026	0.049	0.955	0.171	0.262	0.337	0.347
**MCAPL**	**0.234**	0.015	0.295	0.920	0.154	0.303	0.336	0.347
**MCAPR**	**0.286**	0.005	0.337	0.920	0.210	0.205	0.389	0.267
**MCATL**	**0.256**	0.009	0.140	0.920	0.241	0.205	0.260	0.472
**MCATR**	**0.278**	0.005	0.105	0.920	0.215	0.205	0.416	0.234
**MCAOL**	**0.268**	0.007	0.152	0.920	0.217	0.205	0.429	0.234
**MCAOR**	**0.323**	<0.001	0.048	0.955	0.259	0.182	0.530	0.104
**MCAIL**	**0.292**	0.005	0.307	0.920	0.278	0.169	0.247	0.472
**MCAIR**	**0.316**	<0.001	0.210	0.920	0.313	0.130	0.283	0.472

*Note*: Linear regression analysis was applied to investigate the association between QTM velocity and the ALPS index, adjusting for age, sex, and education.

Abbreviations: AD, Alzheimer's disease; ALPS, analysis along the perivascular space; LLSL, lateral lenticulostriate left; LLSR, lateral lenticulostriate right; MCAFL, frontal pars of middle cerebral artery left; MCAFR, frontal pars of middle cerebral artery right; MCAIL, insular pars of middle cerebral artery left; MCAIR, insular pars of middle cerebral artery right; MCAOL, occipital pars of middle cerebral artery left; MCAOR, occipital pars of middle cerebral artery right; MCAPL, parietal pars of middle cerebral artery left; MCAPR, parietal pars of middle cerebral artery right; MCATL, temporal pars of middle cerebral artery left; MCATR, temporal pars of middle cerebral artery right; MCI, mild cognitive impairment; NC, normal cognition; QTM, quantitative transport mapping.

## 4 DISCUSSION

We investigated changes in perfusion velocity pattern during AD progression using GCA, as measured by our novel QTM velocity method with mPLD‐ASL MRI. The novelty and contributions of this work are 2‐fold: (1) we found the QTM velocity patterns across the AD continuum; (2) we identified the brain arterial pathways that supplied blood to the earliest altered regions. The results show that reduced perfusion velocity occurs first mainly in the MCAFR and MCATL regions, supplied by the MCA, before spreading to other regions. Group comparisons of QTM velocity and sliding‐window GCA demonstrated that MCATL exhibits reduced QTM velocity in the early stages, and its change drives QTM velocity reductions in several other regions, including ACAL, LLSR, MCAFL, MCATR, MCAOR, MCAIL, MCIIR, PCATL, PCATR, PCAOR, and ACTPR. Additionally, our results reveal that QTM velocity in both the MCAFR and MCATL regions is significantly associated with overall cognition (MMSE), immediate recall score, TMT‐A, TMT‐B, and VFT.

### 4.1 Blood perfusion velocity alteration in MCATL and cognition

The MCATL, supplied by the MCA, includes the superior and middle temporal lobes (MTL), hippocampus, parahippocampal gyrus, and amygdala. The MTL is a critical region for early tau pathology in AD,^42^ where tau deposition occurs many years before Aβ accumulation and serves as a harbinger of future neocortical tau accumulation.^43^ Additionally, brain atrophy in AD has been linked to cognitive decline.^44^ The hippocampus is a key structure of the limbic system, playing a central role in cognitive function. Previous studies have demonstrated that decreased CBF in the hippocampus is associated with lower MMSE scores, as well as lower immediate and delayed recall scores.^4^, ^6^, ^45^ Blood perfusion decline is an early event in AD that precedes brain atrophy and cognitive decline.^46^ We found QTM velocity alteration in the MCATL region during the early stage of AD, which is in line with the previous study.^9^ The QTM velocity change in the MCATL region drove its change in 11 other regions, suggesting a potential MCA vascular pathology in the early stage of AD and providing the pattern of QTM velocity change throughout the progression of AD. QTM velocity in MCATL was correlated with cognition performance in the whole cohort, including global cognition, short‐term memory, executive function, and language. This finding aligns with a previous report suggesting that vessel occlusion in the MCA could disrupt blood flow to the hippocampus. After excluding the AD group from the analysis, most significant correlations disappeared, but the association between QTM velocity in the MCATL and short‐term memory remained significant. Short‐term memory loss is the initial and most common presenting symptom of typical AD. Therefore, QTM velocity in the MCATL has significant potential as a marker for indicating AD progression during its early stages.

### 4.2 Brain blood perfusion velocity alteration pattern in AD progression

Given that vascular injury is an early precursor to reduced cerebral perfusion in AD, we divided the cerebrum into 26 regions (13 per hemisphere) based on cerebral arterial territories.^47^ Our results showed significant QTM velocity differences between the MCI and NC groups in the MCA (MCATL, MCATR, MCAOL, and MCAOR) and PCA (PCATL, PCATR, and PCAOR) territories, while no significant differences were observed in the ACA and VB territories. These findings suggest that blood perfusion velocity changes follow different patterns across regions during AD progression, with reduced cerebral QTM velocity first appearing in the temporal and occipital regions fed by the MCA and PCA. This finding aligns with previous studies, which report that AD pathology, including Aβ and tau deposition,^43^, ^48^ and brain atrophy, initially occurs in the temporal regions.^49^, ^50^ Based on these observations, QTM velocity, as a measure of perfusion velocity in the temporal region, shows potential as an early biomarker for AD diagnosis.

### 4.3 GCA and sliding window for QTM velocity

In this study, we constructed pseudo‐time series of QTM velocity based on cognition scores to explore the relationship between QTM velocity changes in various brain regions during AD progression. Cognition partially reflects AD progression, but there are discrepancies between AD pathology and cognition measures.^51^ In sliding‐window GCA, our results reveal that QTM velocity alteration in MCATL drives QTM velocity changes in PCATR, PCAOL, PCAOR, ACTPL, and ACTPR during the early stage of AD (MMSE scores ≥ 26). QTM velocity alteration in MCAFR only drives its changes in other regions during the progression from moderate to severe AD. These findings demonstrate that MCATL serves as a driving core for QTM velocity changes during the early stage of AD, consistent with a previous longitudinal CBF study in AD.^44^

For sliding‐window analysis, the window length is a critical parameter. A shorter window length provides higher temporal resolution across AD progression, but obtaining accurate GCA coefficients becomes challenging due to limited time points within shorter windows. A previous study demonstrated that sliding window lengths of 30, 50, or 70 yield highly consistent results.^11^ Therefore, a window length of 50 was selected in the current study to balance temporal resolution and the accuracy of GCA coefficients.

### 4.4 Blood perfusion velocity and ISF diffusivity

There is no association between QTM velocity and the ALPS index in the NC, MCI, or AD groups, but a weak correlation was observed in the whole cohort. This suggests that blood perfusion velocity might be partially driven by ISF diffusivity overall, although there may be a complex interplay between them within each diagnostic group. Glymphatic function has been proposed to explain brain clearance deficits in AD. The DTI‐based ALPS index is an effective marker of ISF dynamics, potentially related to glymphatic function.^20^, ^52^, ^53^ QTM velocity reflects blood perfusion in the brain, but may also include partial information on tracer transport from the vascular compartment to the interstitial space, given that the shortest PLD is 0.5 seconds.^54^ Thus, QTM velocity may reflect both blood flow in vascular space and fluid flow in perivascular and interstitial spaces, as the QTM model does not separate neurofluids based on their compartmental distribution.^16^, ^18^ Therefore, investigating the association between the ALPS index and QTM velocity may help elucidate the interaction between the blood perfusion velocity and the ISF dynamics during AD progression, a relationship previously observed in animal models.^18^

### 4.5 Perfusion velocity and blood flow

Traditional arterial input function (AIF)‐based CBF quantification assumes a single global AIF for all voxels within the imaging volume, which can introduce errors and violate the principle of local mass conservation.^3^ Cross‐sectional studies have demonstrated that lower AIF‐based CBF correlates with an increased risk of dementia during normal aging.^55^ However, some studies have failed to demonstrate significant decreases in AIF‐based CBF in patients with MCI compared to NC.^4^, ^6^ These inconsistent findings demonstrate that traditional AIF‐based CBF quantification struggles to detect the trajectory of perfusion changes throughout AD progression. QTM has been shown to provide higher accuracy in quantifying blood perfusion,^3^ and our data have demonstrated that QTM is more sensitive than CBF in detecting perfusion alterations in the early stage of AD (Table S3 in supporting information). Previous studies have shown that QTM velocity has significant value in identifying breast cancer malignancy,^35^ analyzing nasopharyngeal cancer gene expressions,^56^ quantifying lung shunt fraction,^57^ and staging progressive liver disease.^34^

Brain perfusion is closely linked to neuronal activity through neurovascular coupling, ensuring the delivery of oxygen and nutrients based on metabolic demands. However, systemic factors, including blood pressure, vascular health, and the autoregulatory capacity of blood vessels, also play significant roles. In the early stages of AD, a mismatch between brain perfusion and blood velocity may indicate compensatory mechanisms within CBF regulation, particularly involving neurovascular coupling.^4^ The brain's autoregulatory system maintains adequate perfusion by adjusting vessel diameter, even under conditions of vascular dysfunction. This compensation can mask early deficits in AIF‐based CBF quantification. In contrast, blood perfusion velocity is less buffered by these regulatory mechanisms and may be more effective in detecting early perfusion changes. These support our finding that QTM velocity is more sensitive than conventional CBF measures in identifying early perfusion changes in AD. Furthermore, it highlights QTM velocity as a promising indicator of disease progression, particularly during the early stages.

### 4.6 Limitations of this study

Several limitations of this study should be acknowledged. First, the sample sizes across diagnostic groups are unbalanced, with relatively small sample sizes in the NC and AD groups. A large‐scale prospective study, including participants with subjective cognitive decline, is needed to further explore the Granger causality of blood flow velocity changes. Second, amyloid positron emission tomography (PET) imaging data were not available, and thus the association between QTM velocity, brain clearance, and Aβ deposition could not be examined. Third, GCA was applied to a cognitive score–ordered cross‐sectional dataset and does not support temporal inference. Moreover, the MMSE and RAVLT were coarse, non‐linear, and potentially noisy and thus could not completely capture AD pathology, which might not fully satisfy the condition of using GCA. Fourth, although we have done the numerical validation in simulated microvasculature of the kidney,^3^ the porous medium theory used in the simulations in kidney tissue or numerical phantoms does not substitute for empirical validation against accepted cerebral perfusion. Given the extremely dense microvasculature in the brain cortex, direct physiological validations of brain voxel‐wise QTM velocity with conventional blood velocity measured by Doppler,^58^ phase‐contrast MRI,^59^ or perfusion PET^60^ methods are challenging. Future work for physiological validation might be using large‐scale microvascular simulation in the brain voxel with deep learning networks.^61^ The near future work might be to cross‐validate QTM velocity with CBF and cerebral blood volume (CBV) to see their physiological relationships and understand the meaning of QTM velocity. Finally, fluid flow in perivascular space was not included due to the complexity of measuring it with MRI, and thus the association between QTM velocity and ISF flow in perivascular space could not be investigated. Future studies should focus on resolving the above limitations and validating the findings in this study.

## 5 CONCLUSION

In this study, we found that reduced cerebral QTM velocity initially appears in the occipital and temporal regions supplied by the MCA and PCA. By combining QTM and GCA, we showed that the reduced QTM velocity in the MCATL derives regional QTM velocity reductions during the early stage of AD, indicating its potential as an early biomarker for AD diagnosis. There was a weak association between QTM velocity and the ALPS index in the whole cohort, suggesting that blood perfusion velocity may partially drive the ISF dynamics and glymphatic activities.

## CONFLICT OF INTEREST STATEMENT

Y.W. owns equity of Medimagemetric LLC. G.C. receives consulting fees from Life Molecular Imaging and research funds from Minoryx Therapeutics. All authors declare no other conflicts of interest. Author disclosures are available in the supporting information.

## CONSENT STATEMENT

All human subjects provided informed consent.

## Supporting information



Supporting Information

Supporting Information
