# Supplementary material for: Decreased quantitative transport mapping velocity in the middle cerebral artery–supplied temporal lobe in Alzheimer's disease
Source: Alzheimers Dement. 2025 Jul 29;21(8):e70540. doi: 10.1002/alz.70540 (PMC12307133; doi:10.1002/alz.70540)
Supplement: Supplementary file 1 — Supporting Information [file ALZ-21-e70540-s001.docx]

# Cerebral arterial territories and AAL template

The atlas of brain arterial territories is based on lesion distributions in 1298 acute stroke patients, including 26 cerebral arterial territories and 4 cerebellar territories [1]. AAL template divides the cerebral brain into 90 regions according to high-resolution T1-weighted volume data [2]. For cerebral arterial territories, there are 4 anterior cerebral artery (ACA) regions, 12 middle cerebral artery (MCA) regions, 8 posterior cerebral artery (PCA) regions, and 2 vertebrobasilar artery (VB) regions, as shown in **Supplementary Table 1**.

MCATL territory was identified as an early region of blood perfusion velocity changes transition from NC to MCI. Thus, we investigated the group differences of the QTM velocity in the subregions of MCATL corresponding to the AAL atlas brain regions. QTM velocity values were compared among diagnostic groups using a one-way analysis of covariance with age, sex, and years of education as covariates. The results are shown in **Supplementary Table 2**.

**Supplementary Table 1.** The abbreviation of 26 cerebral arterial territories, their feeding artery, and corresponding AAL brain regions.

| Index | Brain regions | Abbreviation | Major artery | AAL brain regions |
| --- | --- | --- | --- | --- |
| 1 | anterior cerebral artery left | ACAL | ACA | 1,3,5,7,9,19,21,23,25,27,31,33,35,45,49,57,59,61,67,69,71 |
| 2 | anterior cerebral artery right | ACAR | ACA | 2,4,6,8,10,20,22,24,26,28,32,34,36,46,50,58,60,62,68,70,72 |
| 3 | medial lenticulostriate left | MLSL | ACA | 21,31,71 |
| 4 | medial lenticulostriate right | MLSR | ACA | 22,32,72 |
| 5 | lateral lenticulostriate left | LLSL | MCA | 21,29,41,71,73,75,77,79 |
| 6 | lateral lenticulostriate right | LLSR | MCA | 22,30,42,72,74,76,78,80 |
| 7 | frontal pars of middle cerebral artery left | MCAFL | MCA | 1,3,5,7,9,11,13,15,17,21,29,39,57,71,73,79,81,83 |
| 8 | frontal pars of middle cerebral artery right | MCAFR | MCA | 2,4,6,8,10,12,14,16,18,22,30,40,58,72,74,80,82,84 |
| 9 | parietal pars of middle cerebral artery left | MCAPL | MCA | 1,17,29,49,51,57,59,61,63,65,79,81,85 |
| 10 | parietal pars of middle cerebral artery right | MCAPR | MCA | 2,18,30,50,52,58,60,62,64,66,80,82,86 |
| 11 | temporal pars of middle cerebral artery left | MCATL | MCA | 15,17,29,37,39,41,55,63,73,79,81,83,85,87,89 |
| 12 | temporal pars of middle cerebral artery right | MCATR | MCA | 16,18,30,38,40,42,56,64,74,80,82,84,86,88,90 |
| 13 | occipital pars of middle cerebral artery left | MCAOL | MCA | 49,51,53,65,85,89 |
| 14 | occipital pars of middle cerebral artery right | MCAOR | MCA | 50,52,54,66,86,90 |
| 15 | insular pars of middle cerebral artery left | MCAIL | MCA | 17, 21,29,41,73,79,81,83 |
| 16 | insular pars of middle cerebral artery right | MCAIR | MCA | 18,22,30,42,74,80,82,84 |
| 17 | temporal pars of posterior cerebral artery left | PCATL | PCA | 39,47,53,55,85,89 |
| 18 | temporal pars of posterior cerebral artery right | PCATR | PCA | 40,48,54,56,86,90 |
| 19 | occipital pars of posterior cerebral artery left | PCAOL | PCA | 35,37,39,43,45,47,49,51,53,55,59,67,89 |
| 20 | occipital pars of posterior cerebral artery right | PCAOR | PCA | 36,38,40,44,46,48,50,52,54,56,60,68,90 |
| 21 | posterior choroidal and thalamoperfurators left | PCTPL | PCA | 37,39,47,67,71,77 |
| 22 | posterior choroidal and thalamoperfurators right | PCTPR | PCA | 38,40,48,68,72,78 |
| 23 | anterior choroidal and thalamoperfurators left | ACTPL | PCA | 37,39,47,55 |
| 24 | anterior choroidal and thalamoperfurators right | ACTPR | PCA | 38,40,48,56 |
| 25 | basilar left | BL | VB | -- |
| 26 | basilar right | BR | VB | -- |

ACA: anterior cerebral artery; MCA: middle cerebral artery; PCA: posterior cerebral artery; VB: Vertebrobasilar artery.

**Supplementary Table 2.** The group differences of the QTM velocity in the subregions of MCATL, which was divided by the Anatomical Automatic Labeling (AAL) atlas.

| **AAL brain regions** | **NC** | **MCI** | **AD** | **F value** | **p**  **value** |
| --- | --- | --- | --- | --- | --- |
| Frontal_Inf_Orb_L | 8.46±1.93 | 8.13±1.63 | 7.41±2.13 | 1.200 | 0.304 |
| Rolandic_Oper_L | 12.58±2.96 | 11.93±2.90 | 9.83±3.57 | 5.034 | **0.008** |
| Insula_L | 9.70±2.20 | 9.25±1.99 | 8.30±2.73 | 2.247 | 0.110 |
| Hippocampus_L | 8.47±2.13 | 7.75±2.05 | 6.88±1.97 | 2.760 | 0.067 |
| ParaHippocampal_L | 6.85±2.02 | 6.06±1.62 | 5.29±1.55 | 2.995 | 0.053 |
| Amygdala_L | 10.36±2.73 | 9.18±2.33 | 8.30±2.54 | 3.381 | **0.037** |
| Fusiform_L | 4.68±1.84 | 4.27±1.53 | 4.02±1.22 | 0.480 | 0.620 |
| SupraMarginal_L | 12.42±3.41 | 10.84±3.09 | 8.93±3.60 | 7.251 | **0.001** |
| Putamen_L | 10.22±2.51 | 9.25±2.77 | 8.84±3.08 | 1.595 | 0.207 |
| Heschl_L | 12.57±3.36 | 11.53±2.81 | 9.70±3.71 | 4.157 | **0.018** |
| Temporal_Sup_L | 12.59±2.88 | 11.45±2.56 | 9.57±3.54 | 6.514 | **0.002** |
| Temporal_Pole_Sup_L | 8.44±1.63 | 8.05±1.63 | 7.22±1.96 | 4.172 | **0.017** |
| Temporal_Mid_L | 10.66±2.48 | 9.18±2.32 | 7.85±2.85 | 7.615 | **0.001** |
| Temporal_Pole_Mid_L | 6.71±1.50 | 6.28±1.47 | 5.70±1.46 | 3.272 | **0.041** |
| Temporal_Inf_L | 6.30±1.51 | 5.39±1.16 | 5.40±1.58 | 3.539 | **0.032** |

NC: normal cognitive; MCI: mild cognitive impairment; AD: Alzheimer’s disease Dementia.

# Sensitivity analysis of QTM velocity and CBF in differentiating MCI from NC

CBF maps were reconstructed from the mPLD PCASL data using the *oxford_asl* command in BASIL tools included in FSL. The details were described in our previous study [3]. CBF normalization and segmentation are the same as those performed on QTM velocity. The diagnostic performances of CBF and QTM in differentiating MCI from NC were evaluated using receiver operating characteristic (ROC) curve analysis. The ROC analysis was performed on 12 ROIs (MCATL and its driving 11 regions), in which QTM velocity alteration was identified in the early stage of AD. A paired-sample t-test was applied to compare the diagnostic performance between CBF and QTM. The area under the ROC curve (AUC) was summarized in **Supplementary Table 3**. The results showed that 8 regional QTM and only one regional CBF showed significant diagnostic efficiency in differentiating MCI from NC. The mean AUC of regional QTM (mean AUC = 0.639) was significantly higher than that of regional CBF (mean AUC = 0.585) in differentiation between MCI and NC (p = 0.001). These findings demonstrated that QTM is more sensitive than CBF in detecting perfusion changes at the early stage of AD.

**Supplementary Table 3.** Comparison of diagnostic performance between regional QTM and CBF in differentiating MCI from NC using ROC analysis.

| **Region** | **QTM** | | **CBF** | |
| --- | --- | --- | --- | --- |
|  | **AUC** | **p value** | **AUC** | **p value** |
| **ACAL** | 0.604 | 0.0850 | 0.618 | 0.0530 |
| **LLSR** | 0.597 | 0.0979 | 0.518 | 0.7623 |
| **MCAFL** | 0.625 | **0.0399** | 0.612 | 0.0643 |
| **MCATL** | 0.655 | **0.0104** | 0.593 | 0.1353 |
| **MCATR** | 0.637 | **0.0293** | **0.620** | **0.0371** |
| **MCAOR** | 0.597 | 0.1061 | 0.585 | 0.1061 |
| **MCAIL** | 0.672 | **0.0043** | 0.614 | 0.0868 |
| **MCAIR** | **0.701** | **0.0002** | 0.592 | 0.1400 |
| **PCATL** | 0.661 | **0.0047** | 0.572 | 0.2543 |
| **PCATR** | 0.676 | **0.0011** | 0.551 | 0.3942 |
| **PCAOR** | 0.587 | 0.1765 | 0.553 | 0.4011 |
| **ACTPR** | 0.665 | **0.0060** | 0.597 | 0.1164 |
| **Mean** | **0.639** |  | **0.585** |  |
| **p value** | **0.001** |  |  |  |

AUC: area under the ROC curve.

# References

1. Liu C-F, Hsu J, Xu X, et al (2023) Digital 3D Brain MRI Arterial Territories Atlas. Sci Data 10:74. https://doi.org/10.1038/s41597-022-01923-0

2. Tzourio-Mazoyer N, Landeau B, Papathanassiou D, et al (2002) Automated Anatomical Labeling of Activations in SPM Using a Macroscopic Anatomical Parcellation of the MNI MRI Single-Subject Brain. NeuroImage 15:273–289. https://doi.org/10.1006/nimg.2001.0978

3. Guo Y, Zhou L, Li Y, et al (2024) Quantitative transport mapping of multi-delay arterial spin labeling MRI detects early blood perfusion alterations in Alzheimer’s disease. Alz Res Therapy 16:156. https://doi.org/10.1186/s13195-024-01524-6
